# Blocking soluble Fas Ligand ameliorates pemphigus: PC111 efficacy in ex-vivo human pemphigus models

**DOI:** 10.3389/fimmu.2023.1193032

**Published:** 2023-07-12

**Authors:** Roberta Lotti, Jennifer E. Hundt, Ralf J. Ludwig, Christoph M. Hammers, Brydon Bennett, Antonino Amato, Alessandra Marconi, Carlo Pincelli

**Affiliations:** ^1^ DermoLab, Department of Surgical, Medical, Dental and Morphological Sciences, University of Modena and Reggio Emilia, Modena, Italy; ^2^ PinCell s.r.l., Milan, Italy; ^3^ Lübeck Institute of Experimental Dermatology, University of Lübeck, Lubeck, Germany

**Keywords:** pemphigus, FasL, acantholysis, drug development, ex-vivo model

## Abstract

Pemphigus is a life-threatening, chronic, autoimmune bullous disease affecting both the skin and the mucous membranes. Based on the mainstream concept that blister formation occurs upon binding of autoantibodies to their antigen proteins (desmoglein1, DSG1 and desmoglein3, DSG3), current therapies mostly aim to suppress the immune system. To avoid the severe side effects associated with the chronic use of immunosuppressive treatments, we have developed PC111, a fully human monoclonal antibody targeting human Fas ligand (FasL). We have provided a number of *in vitro* and *in vivo* evidences showing that soluble FasL induces keratinocyte apoptosis followed by acantholysis. An anti-murine FasL prevents blister formation in the pemphigus neonatal mouse model. To confirm the mechanism of action (MoA) and the efficacy of PC111 in a human pemphigus context, we used the keratinocyte dissociation assay and two independent Human Skin Organ Cultures (HSOC) pemphigus models. PC111 reduced acantholysis *in vitro*, as shown by the dose-dependent reduction of fragments in the monolayer cultures. In the first HSOC model, normal human skin was subcutaneously injected with a scFv antibody fragment directed against DSG1 and DSG3, resulting in a severe acantholysis (70-100%) after 24 hours. PC111 inhibited blister formation to around 50% of control. In the second model, normal human skin was injected with a mixture of pemphigus patients’ autoantibodies resulting in a less severe acantholysis (20-30%). PC111 significantly suppressed blister formation to more than 75% up to 72 hours. These results confirm PC111 MoA and demonstrates the efficacy of the anti-FasL antibody also in a pemphigus setting.

## 1 Introduction

Pemphigus is a chronic, autoimmune blistering disease, characterized by loss of keratinocyte adhesion (acantholysis) leading to the formation of the blisters. Pemphigus affects both the skin and the mucous membranes where flaccid bullae and erosions are normally observed. Patients’ autoantibodies (generally named PVIgG) binding to the main desmosome target antigens (DSG1 and DSG3) has long been considered to directly induce blister formation (1). Yet, it has now become clear that additional mechanisms following antibody binding contribute to skin blistering in pemphigus. Pathogenic IgGs activate phosphatidylcholine specific phospholipase C (PLC), which in turn elevates intracellular free calcium, and activates various kinases including p38MAP Kinase, Akt, Src, epidermal growth factor receptor kinase (EFGRK), and protein kinase C (PKC) (2, 3). Moreover, several lines of evidence indicate that keratinocyte apoptosis is involved in the pathological mechanisms of pemphigus [reviewed by Grando et al. (4)]. In particular, cleaved caspase-8 and -3 are detected in keratinocytes of pemphigus lesions, and caspase-8-positive keratinocytes display increased Fas Ligand expression (5).

Fas Ligand is a transmembrane protein (mFasL) that can be proteolytically cleaved to generate its soluble form of 26 kDa (sFasL) (6). Both forms of FasL can bind to their receptor, Fas, also known as CD95 or Apo1. FasL is normally stored within intracellular secretion vesicles, which, upon a certain stimulus (i.e. recognition of a target cells in the case of T cells and NK), fuse to the cell membrane resulting in the surface expression of FasL (7). Binding of mFasL to Fas receptor triggers the extrinsic apoptotic pathway through the activation of caspase-8 (8). On the other hand, sFasL appears to have both non-apoptotic and pro-inflammatory activities in immune cells (9, 10). In healthy skin, FasL is homogeneously stored in vesicles within the cytoplasm, in association to intermediate filaments of keratinocytes localized in the basal and first suprabasal layers of the epidermis (11). It has been reported that PVIgG are able to upregulate FasL expression, at the mRNA and protein level, and increase its translocation to the plasma membrane of human keratinocytes (12). Accordingly, sera from pemphigus patients contain abnormally elevated levels of sFasL (13). Anti-FasL antibodies (Ab) prevent PVIgG-induced caspase-8 activation and DSG cleavage in human keratinocytes *in vitro* (4) as well as blister formation in an *in vivo* mouse model of pemphigus (14). Finally, strong evidence of the critical role of sFasL in blister formation in pemphigus has been shown using two mutant mice selectively lacking either sFasL (FasLΔs/Δs) or mFasL (FasLΔm/Δm). Indeed, only the FasLΔs/Δs animals showed negligible levels of acantholysis induced by PVIgG injection, clearly indicating that soluble FasL, and not the membrane form, plays a crucial role in the mechanism of blister formation (14).

Currently, pemphigus therapy is based on chronic and systemic immunosuppression that is often associated with severe adverse events. Therefore, there is still need for novel treatments directed to altered pathways downstream of the antibody binding to the target antigens. Given the critical role of FasL in the signaling leading to blister formation in pemphigus, we have developed PC111, a fully human monoclonal antibody directed against the soluble form of FasL. Despite the body of evidence on the activity of anti-FasL Ab, we wanted to confirm the mechanism of action and the efficacy of PC111 in human contexts. To this purpose, we have used the *in vitro* keratinocyte dissociation assay and two 3D ex-vivo models, showing that neutralizing human FasL blocks acantholysis.

## 2 Materials and methods

### 2.1 Human biomaterial collection

All experiments with human samples were approved either by the Ethical Committee of Area Vasta Emilia Nord Policlinico of Modena, Italy (Protocol number 1186/2019) or by the Ethical Committee of the Medical Faculty of the University of Lubeck (ref number: 12-178/06-109). All subjects gave written informed consent in accordance with the Declaration of Helsinki. For sera collection, patients with suspected autoimmune bullous disease were enrolled during their first access to the Dermatology Unit, Rare Disease Room, of the Policlinico of Modena. Diagnosis of pemphigus was confirmed by routine laboratory tests (Indirect immunofluorescence and DSG ELISA, MBL International Corp., Nagoya, Japan). Patients negative for the diagnosis of pemphigus or any other skin bullous autoimmune disease were inserted in negative control group. Sera were then taken from the renaming volume after the routine diagnosis. For this study, we enrolled a total of 15 patients with pemphigus (PV, mucocutaneous pemphigus, and PF) and 8 negative patients.

Healthy human skin was obtained from discarded material of healthy patients undergoing cosmetic plastic surgery, usually for abdominal or breast reduction.

### 2.2 Reagent preparation

The HA-tagged scFv directed against DSG1 and DSG3 (also termed Px4-3) (15, 16) was produced in *Escherichia Coli* and purified as previously described (15).

Pemphigus autoantibodies (generally named PVIgG) and normal human IgG (NIgG) were purified by affinity binding on a HiTrapProtein G HP column (GE Healthcare Bio-Science, Piscataway, NJ, USA) as previously described (14). Sera were pooled and loaded on the column after dilution in binding buffer. Bound IgG was eluted with elution buffer (0.1 M glycine/HCl, pH 2.7) and immediately neutralized by Tris 1 M (pH 9). After dialysis against PBS and concentration by ultrafiltration (using Amicon, Beverly, MA, USA), IgG were filter-sterilized, and stored at +4°C until use. Protein concentration was determined by Bradford assay using protein Standard I (BioRad, Hercules, CA, USA). In general, PVIgG were purified from a mix of patients’ sera collected from patients with PV, mucocutaneous pemphigus, and PF and pooled for IgG purification (ELISA titers of pooled samples: DSG1: 116.06 U/ml; DSG3: 179.27 U/ml);, while NIgG were isolated from sera from control patients (ELISA titers of pooled samples: DSG1: 5.05 U/ml; DSG3: 3.15 U/ml).

### 2.3 Keratinocyte dissociation assay

Primary cultured normal human keratinocytes (NHK) were obtained and expanded as previously described (14). Keratinocytes were seeded in 12-wells plate and maintained in a defined serum-free medium (KGM, Lonza Walkersville Inc., Walkersville, MD, USA). When cells reached 100% confluency, the calcium concentration was switched to 1.8 mM for at least 24 hours, to induce keratinocyte differentiation and desmosome maturation. NIgG or PVIgG were added at a concentration of 2 mg/ml in KBM. PVIgG were also used in combination with increasing doses of PC111 (from 0.001 to10 μg/ml) administered 2 hrs after patients’ IgG and incubated at 37°C for 72 hours. As a control, we also used NIgG + PC111 at the highest dose 10 μg/ml. NHK were then incubated with dispase II (>2.4 U/ml; Roche®, Basel, Switzerland) to release monolayers that were in turn exposed to mechanical stress induced by repeated pipetting. Fragments were then fixed and stained with 1% Rhodamine B solution (Sigma‐Aldrich, Taufkirchen, Germany). Dissociation score was calculated per each sample applying the formula below, considering N as the number of cell sheets in each well:


$$Dissociation\;Score=\frac{N-N\text{NIgG}}{N\text{PVIgG}-N\text{NIgG}}\times 100$$

### 2.4 scFv-induced pemphigus human skin organ culture model

The protocol used for the scFv-induced pemphigus HSOC has previously been described in detail (17). Briefly, healthy human skin (used within 24 h after collection) was cut into 1cm^2^ sections and stored in William’s E medium (Gibco) on ice until further use. Defined keratinocyte serum-free medium (D-K-SFM; Gibco) was added to the wells of a transwell cell culture insert plate (Sigma-Aldrich) maintaining the air-liquid interface between the epidermis and the air. To induce acantholysis, a human single-chain variable fragment (scFv) directed against DSG3 and DSG1 (also termed Px4-3) was used, while human intravenous immunoglobulins (IVIgG) or DPBS were injected as a negative control. The scFv was injected at a concentration of 3.75 µg/µl. PC111 was subcutaneously (s.c.) injected at 3 different concentrations (1, 10, or 50 µg) in a total volume of 50 µl 2 hours after scFv injection. Skin specimens were allocated by randomization to the different groups. Afterwards, the transwell plates were maintained in the incubator for 24 hours. Skin samples were then harvested and fixed in 4% Histofix solution for subsequent paraffin embedding and hematoxylin eosin (H&E) staining. Slices (5–10) were analyzed for each sample in order to cover the entire skin sample.

### 2.5 PVIgG-induced pemphigus human skin organ culture model

A second pemphigus HSOC model was set up, based on the model published by Egu and co-workers (18), using purified pemphigus antibodies mixture (PVIgG). Healthy human skin (<24 hours post-surgery) was cut into cubic sections of 0.5 x 0.5 cm. Skin specimens were allocated by randomization to the different groups. Purified PVIgG or NIgG were injected s.c. into the dermis of each tissue cube using an insulin syringe (30G gauge) in a total maximum volume of 50 µl. We tested different concentrations of PVIgG (PV1000: 1000 μg; PV1500: 1500 μg) in order to define the pathogenic concentration able to induce clear acantholysis at the histological level. PC111 was s.c. injected 2 hours after PVIgG treatment at 3 different concentrations (10, 50, or 100 µg). After injection, skin specimens were kept in organ culture in DMEM + 1% Pen/Strep in a 12 multi-wells plate and harvested after 24- and 72-hours treatment. Samples were then formalin fixed and paraffin embedded for histology analysis. We analyzed all of the skin biopsy by means of sequential cutting, with steps of 200-250 μm between each cut; approximately 8-10 slices were analyzed for each biopsy. Slices were stained by H&E and captured by D-Sight 2.0 software (Menarini Diagnostics, Italy) at 10X magnification for acantholysis evaluation.

### 2.6 Direct immunofluorescence staining

To show the binding of scFv to the treated epidermis, cryosections of scFv-treated HSOC skin pieces were stained using a Rat Anti-HA High-Affinity monoclonal Antibody (Roche). After few washes, samples were incubated with Alexa Fluor 594‐conjugated goat anti‐rat IgG (Life Technologies, Carlsbad, USA). Samples were then mounted in fluoromount-G and coverslips for the analysis. On the other hand, to show the binding of human IgG to the treated epidermis, sections of NIgG- or PVIgG-treated HSOC skin pieces were stained using polyclonal rabbit anti-human IgG/FITC conjugated antibodies (Dako, Glostrup, Denmark). After several washes, samples were mounted in fluoromount-G and coverslips for the analysis. Pictures were captured by ZOE Fluorescent Cell Imager (BioRad).

### 2.7 Acantholysis evaluation

Using the ImageJ software, the percentage of epidermal split formation, identified as areas of the epidermis in which suprabasal cell detachment spreads along more than four adjacent basal cells, was quantified by an investigator blinded to sample treatments. Splits were measured in each visual field as % of the skin coverage with 100% representing acantholysis covering the entire skin section.

### 2.8 Statistical analysis

If not otherwise indicated, data are presented as mean ± SEM. For statistical analysis, we used Prism Software (version 9; GraphPad; San Diego, CA, USA). One-way or two-way ANOVA was used for multiple comparisons. A value of p< 0.05 or less was assumed to indicate a statistically significant difference in the compared parameters. Each statistical analysis performed was detailed in the figure legend.

## 3 Results and discussion

### 3.1 PC111 inhibits human keratinocyte detachment *in vitro*

**Figure 1:**
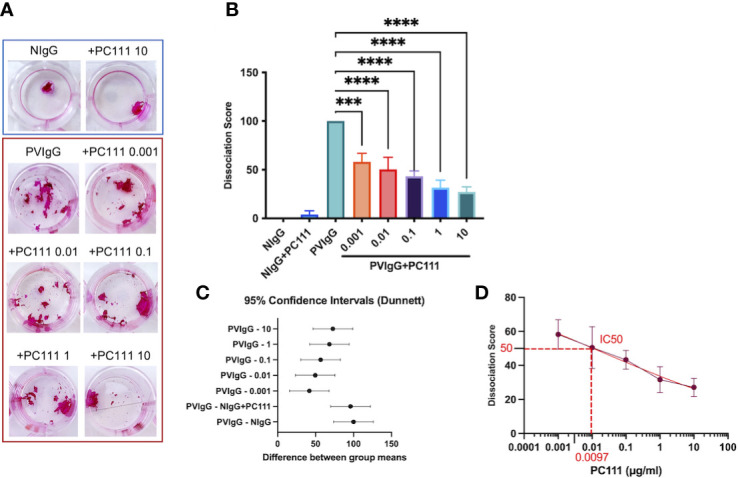
PC111 prevents keratinocyte acantholysis *in vitro*. Keratinocytes were cultured with purified normal human IgG (NIgG) or pemphigus autoantibodies (PVIgG) in the presence or absence of PC111 (from 0.001 to 10 µg/ml), and dispase-based dissociation assay was performed. **(A)** Representative images of cell sheet fragmentation, after staining with Rhodamine B. **(B)** Quantification obtained by counting the number of fragments of cell sheets and represented as dissociation score (means ± SEM). In general P< 0.0001 for ordinary one-way ANOVA for differences among groups; ***P< 0.001 and ****P< 0.0001 for Dunnett’s multiple comparisons test versus PVIgG (n = 5 independent experiments). **(C)** Graph representing differences between group means and 95% Confidence Intervals (according to multiple comparisons with Dunnett analysis). **(D)** Dose response curve with absolute IC50 calculation.

Pemphigus is characterized by keratinocyte detachment at the desmosome level resulting in acantholysis. An *in vitro* keratinocyte dissociation assay was established to evaluate the pathogenicity of pemphigus autoantibodies (19). Using the keratinocyte dissociation assay, we demonstrated that PC111, administered few hours after pemphigus autoantibody stimulus, reduced acantholysis *in vitro*, as shown by the dose-dependent reduction of fragments in the monolayer cultures (**Figure 1A**). This was confirmed by the dissociation score in a statistically significant manner (**Figures 1B, C**). Indeed, both ordinary one-way ANOVA test plus multiple comparison (**Figure 1B**) and the 95% Confidence Interval on Mean differences (Dunnett’s) (**Figure 1C**) highlighted a statistical difference in all PC111-treated groups versus the group stimulated with PVIgG only. We also calculated the IC50 of PC111 and determined that a concentration as low as 0.0097 μg/ml was sufficient to inhibit 50% of acantholysis *in vitro* (**Figure 1D**). No changes in the monolayer cultures were obtained when either NIgG or NigG+PC111 were added to the cells (**Figures 1A, B**). These findings are in line with and further confirm previous findings on the role of FasL in blister formation (14). In addition, pemphigus autoantibodies induce an upregulation of FasL and a release of FasL from keratinocytes upon binding to their antigens (5). Cells silenced for FasL expression are protected from PVIgG-induced acantholysis, and the use of an anti-FasL neutralizing antibody reduces the acantholytic effect *in vitro* (14). Here, we validated the use of the new monoclonal antibody PC111 in a “curative” protocol, in which PC111 displays a very strong effect in the amelioration of blisters *in vitro* till 72 hrs of incubation. Indeed, we used a 72 hrs incubation period, slightly modifying the original method (described in 19), given that our aim was to evaluate the effective and prolonged curative potential of PC111, in an already validated setting (14).

### 3.2 PC111 prevents blister formation in two independent HSOC models

In order to confirm the efficacy of PC111 in a human context, we used two independent protocols of pemphigus in human skin organ culture (HSOC) models. While the startup PinCell was in the process of refining the proof of concept on the effect of PC111 in human pemphigus, Prof. Detlef Zillikens, member of PinCell Scientific Advisory Board, inspired us by strongly recommending the use of 3D ex-vivo human models given that they are optimal complementary tools for *in vivo* pemphigus models (20).

**Figure 2:**
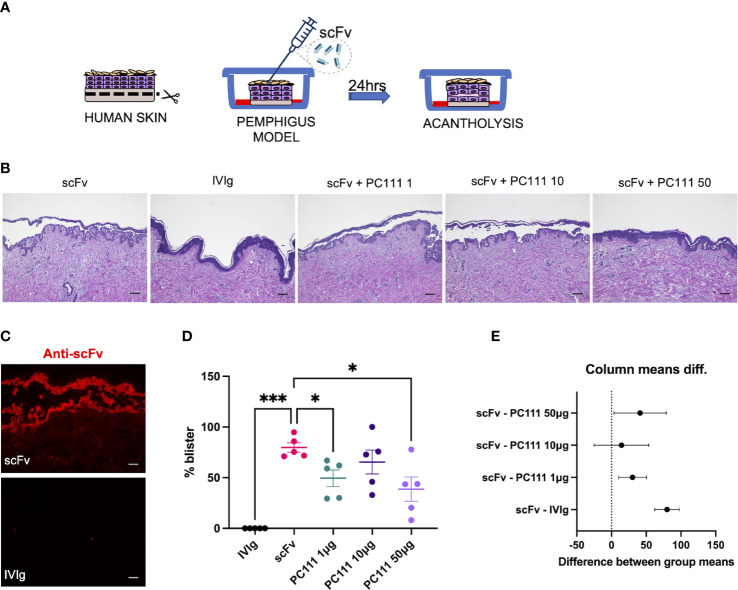
PC111 inhibits blister formation in scFv-induced ex vivo pemphigus model. **(A)** Schematic representation of the method used. **(B)** Exemplary pictures of the H&E staining of the 24 hrs cultures. PC111 (1-10-50 µg) was injected s.c. 2 hrs after scFv. IVIg were used as negative control. Scale bar = 100 μm. **(C)** Pictures showing direct immunofluorescence microscopy against scFv. Scale bar = 50 μm **(D)** Evaluation of the length of the blister expressed as % of the entire skin analyzed. Results are presented as mean ± SEM of 5 different experiments. In general, P =0.0001 for ordinary one-way ANOVA for differences among groups; *P<0.05; ***P< 0.001 for Dunnett’s multiple comparisons test versus scFv. **(E)** Graph representing differences between group means and 95% Confidence Intervals (according to multiple comparisons with Dunnett analysis).

The first model was performed at the University of Lubeck, where the scFv-induced pemphigus HSCO model has been developed. This method was published by the Zillikens’s group from the department of Dermatology in Lubeck a few years ago (17). In this model, normal full thickness human skin was injected s.c. with a pathogenic anti-DSG1 and DSG3 single-chain variable fragment scFv (also formerly named Px4-3, as described in 15 and 16) in a dose able to induce 75-100% acantholysis after 24 hours of treatment (Scheme in **Figure 2A**). IVIg were used as negative control. PC111 was s.c. injected 2 hours after scFv, to mimic a curative and not a preventive treatment, at 3 different doses (1, 10 and 50 μg). After 24 hours, scFv-injected skin samples developed intraepidermal split formation at the suprabasal level, while a substantial inhibition of blister formation was observed with the highest dose of PC111, as shown by H&E staining (**Figure 2B**). By direct immunofluorescence, we effectively demonstrated the deposition of the scFv injected fragments at the inter-keratinocyte level only in scFv-injected skin samples (**Figure 2C**). Measurement of the epidermal splits (blisters) demonstrated that scFv induced a mean of 80% acantholysis, while no acantholytic effect was observed after IVIg injection. PC111 significantly reduced blister formation to around 50% of the scFv-treated controls (**Figure 2D**). We confirmed a statistical difference by calculating the 95% Confidence Interval on Mean differences (Dunnett’s) comparing PC111 treated groups versus scFv alone (**Figure 2E**).

**Figure 3:**
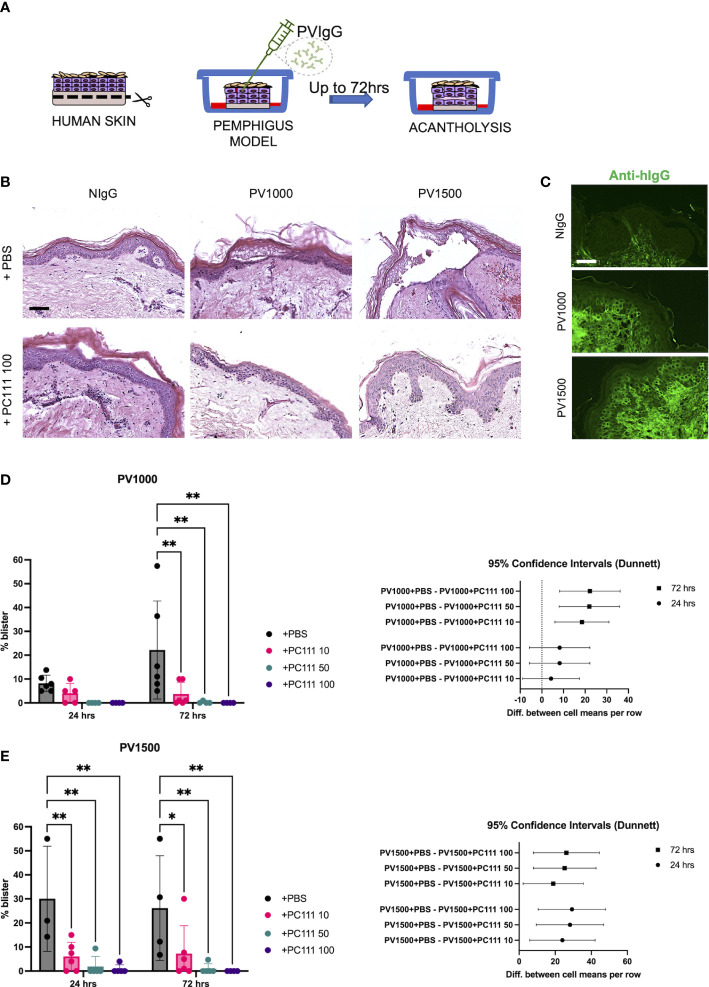
PC111 inhibits blister formation in PVIgG-induced ex vivo pemphigus model. **(A)** Schematic representation of the method used. **(B)** Exemplary pictures of the H&E staining of the 24 hrs cultures of skin injected s.c. with NIgG as negative control (1000 µg), PVIgG 1000 µg (PV1000) or PVIgG 1500 µg (PV1500) injected with diluent (PBS) or treated with PC111 100 µg (PC111 100). Scale bar = 100 μm. **(C)** Representative pictures showing direct immunofluorescence microscopy against hIgG in samples treated with NIgG, PVIgG 1000 µg (PV1000) or PVIgG 1500 µg (PV1500) injected with diluent (PBS). Scale bar = 50 μm. **(D)** On the left, evaluation of the length of the blister induced by PVIgG 1000 µg (PV1000) of samples injected 2hrs after PVIgG with PC111 (10-50-100 µg) expressed as % of the entire skin analyzed. Results are presented as mean ± SEM of 4 different samples. Two-way ANOVA test was followed by multiple comparison analysis. **P<0.01 per Dunnett’s multiple comparisons test versus PVIgG. On the right, graph representing differences between group means at 24 (circles) and 72 (squares) hrs and 95% Confidence Intervals (according to multiple comparisons with Dunnett analysis). **(E)** On the left, evaluation of the length of the blister induced by PVIgG 1500 µg (PV1500) of samples injected 2hrs after PVIgG with PC111 (10-50-100 µg) expressed as % of the entire skin analyzed. Results are presented as mean ± SEM of 4 different samples. Two-way ANOVA test was followed by multiple comparison analysis. *P<0.05 and **P<0.01 per Dunnett’s multiple comparisons test versus PVIgG. On the right, graph representing differences between group means at 24 (circles) and 72 (squares) hrs and 95% Confidence Intervals (according to multiple comparisons with Dunnett analysis).

In the second model (inspired by 18), normal human skin was injected with two different amounts of purified pemphigus IgG mixture (PVIgG: PV1000 and PV1500) directed against both DSG1 and DSG3, and acantholysis was evaluated at 24 and 72 hours (Scheme in **Figure 3A**). Both PV1000 and PV1500 induced split formation at the suprabasal level, as shown by epidermal split in H&E staining, while the addition of PC111 2 hrs after pathogenic IgG injection prevented blister formation (**Figure 3B**). We then confirmed the deposition of the injected pemphigus IgG at the inter-keratinocyte level in both PV1000 and PV1500 treated skin samples (**Figure 3C**). The measurement of split length show that PV1000 induced 10 and 20% acantholysis at 24 and 72 hours, respectively. At 24 hours, PC111 tended to reduce blister formation, but not in a statistically significant manner. On the other hand, at 72 hours, when more severe acantholysis had developed, PC111 treatment induced a dose-dependent and statistically significant inhibition of blisters (**Figure 3D**). At 24 hrs blister formation is mild, possibly due to the fact that more incubation time is needed to induce a frank acantholysis at low concentrations of pemphigus antibodies, as formerly hypothesized by Schiltz and Michel (21). On the other hand, PV1500 induced 30% acantholysis at both 24 and 72 hours. In this context, PC111 significantly reduced blister formation in a dose-dependent manner both at 24 and 72 hours, as confirmed also by the 95% CI analysis (**Figure 3E**). In general, also the negative control sample of HSOC models maintained in culture for up to 72 hrs did not show any alterations (personal observations). These findings are in line with data presented in the pioneer study on ex vivo model of pemphigus performed in 1976 by Schiltz and Michel. Indeed, they demonstrated that the relative incorporation of H3uridine by the control samples was fairly constant during the first 67 hrs in culture. This indicates that our model is valid and reliable.

The MoA of PC111 was confirmed in two different HSOC models in order to reproduce pemphigus with diverse phenotypes and severity. Although we were not able to directly quantify the effective upregulation and release of sFasL in HSOC models, here we indirectly confirmed that the binding of autoantibodies to DSGs induce the release of sFasL from target keratinocytes, as previously postulated (5, 14), also in a more complex system. Our approach will allow a broad use of the new anti-FasL antibody. Indeed, scFv-induced pemphigus seems to reflect an extremely severe kind of pemphigus in humans, characterized by the almost complete detachment of the epidermis, while the PVIgG-induced model recapitulates a milder disease. In any case, the efficacy of PC111 in blocking blister formation was confirmed by two independent laboratories with two different models.

## 4 Conclusions

sFasL plays a critical role in the pathomechanisms underlying the formation of the blister in pemphigus (14, 22). We have been developing a human anti-FasL monoclonal antibody (PC111) that, by targeting sFasL in the skin at the keratinocyte level, downstream of autoantibodies binding to DSGs, does not affect the immune system, thus potentially avoiding the severe side effects associated with immunosuppressive treatments currently used in pemphigus. We had shown previously that FasL siRNA prevented DSG cleavage (14), while an anti-FasL antibody blocked blister formation in the passive neonatal pemphigus mouse model in a dose-dependent manner (14). Recently, using a modified version of the original model by Ohyama and co-workers (23), we have generated an active pemphigus mouse model that allows tracking of disease progression in adult animals for several weeks. The intraperitoneal injection of an anti-FasL antibody significantly reduced PV score up to 8 weeks, while restoring the weight loss and prolonging mice survival (unpublished data). While the proof of concept for FasL inhibition in the treatment of pemphigus is compelling, based on the body of *in vitro* and *in vivo* evidence, the data presented here confirm that the human anti-FasL antibody PC111 blocked blister formation in different human environments. In fact, PC111 inhibited acantholysis in the human keratinocyte dissociation assay and blocked blister formation in two independent and slightly diverse 3D ex-vivo pemphigus skin models (HSOC). Recently, HSOC model was successfully validated for the screening of novel treatment targets for pemphigus by simultaneously injecting scFv and the therapeutic compounds (24). In contrast, in both HSOC experiments presented here, PC111 was injected after the induction of the blisters, reflecting the temporal reality of diagnosis followed by treatment in human pemphigus. While scFv induced a marked and widespread epidermal detachment, mimicking an extremely severe form of pemphigus, PVIgG produced a milder acantholysis, possibly more similar to the pemphigus patients’ situation. Nevertheless, PC111 significantly inhibited blister formation in both HSOC methods under a therapeutic protocol of administration. Currently, the most effective treatments for pemphigus target the production of antibodies by B-cells, such as anti-CD20 Abs, while inhibiting intracellular signaling pathways has not been fully investigated in clinical settings. Anti-B-cell treatment normally takes a few weeks to ameliorate symptoms and is potentially associated with immunosuppression-related side effects. On the other hand, PC111 displays a rapid onset of action, without affecting the immune system, possibly spearing/avoiding the use of steroids.

In conclusion, here we provide further evidence on the efficacy of specifically targeting sFasL in pemphigus and propose PC111 as a potential non-immunosuppressive treatment for this severe and life-threatening disease, opening the way to further development of this approach in clinical settings.

## Data availability statement

The raw data supporting the conclusions of this article will be made available by the authors, without undue reservation.

## Ethics statement

The studies involving human participants were reviewed and approved by Ethic Committee of Area Vasta Emilia Nord Policlinico of Modena, Italy and Ethical Committee of the Medical Faculty of the University of Lubeck. The patients/participants provided their written informed consent to participate in this study.

## Author contributions

RL, BB, JH, AM, and CP contributed to conception and design of the study. RL and JH performed experiments and data collection. JH and RL analysed data. CMH provided scFv. RL performed the statistical analysis. RL, AM, CP, AA, JH and RJL have discussed and interpreted the data. RL and CP wrote the first draft of the manuscript. RJL, BB, AA and AM wrote sections of the manuscript. All authors contributed to the article and approved the submitted version.
