# Corrigendum: Blocking soluble Fas Ligand ameliorates pemphigus: PC111 efficacy in ex-vivo human pemphigus models

**DOI:** 10.3389/fimmu.2024.1331003

**Published:** 2024-01-23

**Authors:** Roberta Lotti, Jennifer E. Hundt, Ralf J. Ludwig, Christoph M. Hammers, Brydon Bennett, Antonino Amato, Alessandra Marconi, Carlo Pincelli

**Affiliations:** ^1^ DermoLab, Department of Surgical, Medical, Dental and Morphological Sciences, University of Modena and Reggio Emilia, Modena, Italy; ^2^ PinCell s.r.l., Milan, Italy; ^3^ Lübeck Institute of Experimental Dermatology, University of Lübeck, Lubeck, Germany

**Keywords:** pemphigus, FasL, acantholysis, drug development, ex-vivo model

In the published article, there was an error in the author list, and author Christoph M. Hammers was erroneously excluded. The corrected author list appears below.

Roberta Lotti^1,2^, Jennifer E. Hundt^3^, Ralf J. Ludwig^3^, Christoph M. Hammers^3^, Brydon Bennett^2^, Antonino Amato^2^, Alessandra Marconi^1,2†^, Carlo Pincelli^1,2†^

A correction has been made to **Author contributions.**

This sentence previously stated:

“RL, BB, JH, AM, and CP contributed to conception and design of the study. RL and JH performed experiments and data collection. JH and RL analysed data. RL performed the statistical analysis. RL, AM, CP, AA, JH and RJL have discussed and interpreted the data. RL and CP wrote the first draft of the manuscript. RJL, BB, AA and AM wrote sections of the manuscript. All authors contributed to the article and approved the submitted version.”

The corrected sentence appears below:

“RL, BB, JH, AM, and CP contributed to conception and design of the study. RL and JH performed experiments and data collection. JH and RL analysed data. CMH provided scFv. RL performed the statistical analysis. RL, AM, CP, AA, JH and RJL have discussed and interpreted the data. RL and CP wrote the first draft of the manuscript. RJL, BB, AA and AM wrote sections of the manuscript. All authors contributed to the article and approved the submitted version.”

The **Acknowledgments**, which previously stated “We thank Christoph M. Hammers from Lübeck Institute of Experimental Dermatology for providing scFv.”, has also been deleted.

The authors apologize for this error and state that this does not change the scientific conclusions of the article in any way. The original article has been updated.

